# The Relationship between the Behavioral Hearing Thresholds and Maximum Bilirubin Levels at Birth in Children with a History of Neonatal Hyperbilirubinemia

**Published:** 2013-06

**Authors:** Rasool Panahi, Zahra Jafari, Abdoreza Sheibanizade, Masoud Salehi, Abdoreza Esteghamati, Sara Hasani

**Affiliations:** 1 Department of Audiology, Faculty of Rehabilitation, Tehran University of Medical Sciences, Tehran, Iran.; 2 Department of Basic Sciences in Rehabilitation, Faculty of Rehabilitation, Rehabilitation Research Center, Tehran University of Medical Sciences, Tehran, Iran.; 3 Department of Biostatistics, Faculty of Management and Medical Information, University of Medical Sciences, Tehran, Iran.; 4 Children's Hospital, martyr Akbar-Abadi, Tehran University of Medical Sciences, Tehran, Iran.

**Keywords:** Behavioral, Hearing threshold, Hearing loss, Hyperbilirubinemia, Neonatal

## Abstract

**Introduction:**

Neonatal hyperbilirubinemia is one of the most important factors affecting the auditory system and can cause sensorineural hearing loss. This study investigated the relationship between behavioral hearing thresholds in children with a history of jaundice and the maximum level of bilirubin concentration in the blood.

**Materials and Methods:**

This study was performed on 18 children with a mean age of 5.6 years and with a history of neonatal hyperbilirubinemia. Behavioral hearing thresholds, transient evoked emissions and brainstem evoked responses were evaluated in all children.

**Results:**

Six children (33.3%) had normal hearing thresholds and the remaining (66.7%) had some degree of hearing loss. There was no significant relationship (r=-0.28, P=0.09) between the mean total bilirubin levels and behavioral hearing thresholds in all samples. A transient evoked emission was seen only in children with normal hearing thresholds however in eight cases brainstem evoked responses had not detected.

**Conclusion:**

Increased blood levels of bilirubin at the neonatal period were potentially one of the causes of hearing loss. There was a lack of a direct relationship between neonatal bilirubin levels and the average hearing thresholds which emphasizes on the necessity of monitoring the various amounts of bilirubin levels.

## Introduction

High bilirubin level at birth has been considered as one of the most important factors affecting the auditory system. Although this disease does not have thoughtful consequences in most infants, in the absence of appropriate treatment, high levels of blood serum bilirubin can result in acute encephalopathy and brain damage (1,2). When red blood cells are broken down, unconjugated bilirubin enters into the plasma. Normally, this type of bilirubin molecules link together by liver enzymes and make conjugated bilirubin that the body is able to excrete. However, if the bilirubin does not convert to a conjugated mode, it accumulates in plasma and its concentration increases. With increasing levels of serum bilirubin, the substance passes through the blood brain barrier and enters the central nervous system. Kernicterus is a neurological syndrome that is due to unconjugated bilirubin deposits in brain cells and nuclei (3). Due to the involvement of vestibular nerve, oculomotor nerve, cerebellum and cerebral basal ganglia, patients with kernicterus develop symptoms such as movement disorders, impaired eye movements and hearing loss. The only clinical sign of kernicterus may be permanent sensorineural hearing loss (4,5). Jaundice in the first day following the birth is always pathologic. Likewise, if the maximum level of bilirubin in term neonate exceeds beyond 13 mg/dL, it is considered pathologic (6). Severe hyperbilirubinemia is defined as serum bilirubin levels above 17 mg/dL (7). It seems that auditory brainstem nuclei including the cochlear nuclei, inferior colliculus and superior olivary complex are the most vulnerable parts of the auditory system against high bilirubin concentrations. Damage to these structures can lead to Sensor-Neural Hearing Loss (SNHL) (3,8). 

Studies that reviewed audiological findings in children with a history of neonatal hyperbilirubinemia have primarily used electrophysiological and non-behavioral tests such as auditory brainstem response (ABR). For instance, the study of Jiang et al on infants with a history of neonatal hyperbili- rubinemia revealed that the threshold of ABR response recorded in these infants was significantly increased (9). In the study of Nickisch and colleagues in 2008 on two groups of children with a history of neonatal hyperbilirubinemia, it was reported that 87% of children in the group with serum bilirubin levels greater than 20 mg/dL and 13% of children in the group with serum bilirubin levels between 12-19 mg/dL suffered from particular hearing impairment (10).

Evidence suggests that lower levels of bilirubin may cause minor encephalopathy that known as bilirubin- induced neurological dysfunction (BIND). In this case, the hearing impairment has been reported alone and without any other signs of kernicterus (8). The relationship between peak serum bilirubin levels and behavioral hearing thresholds has not well-defined yet furthermore there is not enough evidence in this field. 

Considering the results of these studies and assuming that different degrees of neonatal hyperbilirubinemia can cause different degrees of hearing impairment, our study investigated the behavioral hearing thresholds in children with a history of neonatal jaundice and reported the frequency of hearing impairment. This study also addressed the relationship between the average hearing thresholds and level of serum bilirubin.

## Materials and Methods

This study was performed from January to June 2012 at the audiology department of faculty of rehabilitation in Tehran University of Medical Sciences. Our study was performed on 18 children aged 2.4 to 11 years (mean 5.6±2.5 years) and with a history of neonatal hyperbilirubinemia. Of these, 10 were girls (55.6%) and 8 (44.4%) were boys. Additional information regarding the children is summarized in Table 1. Blood bilirubin levels at birth for each child were determined according to the medical record. In the inclusion criteria, a history of phototherapy or exchange transfusion, negative family history of hearing loss, no history of respiratory distress, no history of head trauma, epilepsy and ototoxic medications and negative history of other diseases or syndromes affecting the auditory system were met. The selected children were originated from the children's audiology rehabilitation center as well as the Children's Hospital in Tehran. This study was approved by the ethical committee of Tehran University of Medical Sciences.

At the initiation, a case history was obtained for each child. Subsequently, following ensuring of normal middle ear function using a calibrated Interacoustics AZ26 tympanometer, behavioral air conduction hearing thresholds at frequencies of 500, 1000, 2000 and 4000 Hz were recorded using a calibrated Interacoustics AC40 audiometer. Threshold acquisition procedure was different according to the age of the child. In children aged less than 5 years, the conditioned play audiometry (CPA) was used and in children of 5 to 11 years, conventional adult’s audiometry was performed. Visual reinforcement audiometry 

(VRA) was used only in a 2.4 year old child, to gauge the hearing thresholds.According to numerous studies, in cases with neonatal hyperbilirubinemia, hearing impairment was attributed to cochlear and retrocochlear structures (11-15). To investigate the status of cochlea and auditory nerve, we also performed transient evoked otoacoustic emission (TEOAE) and ABR evaluations in all children. TEOAE recordings were conducted using the ILO88 OAE System. The children were in the supine position with closed eyes. To perform recordings, one thousands click stimuli at 80 dB sound pressure level (SPL) in each ear had been presented.Response reproducibility of 70% (or more) was considered as a criterion for the existence of TEOAE response (16). Click evoked auditory brainstem responses were recorded using the Interacoustics ABR system Eclipse EP25, with 15 ms time window, click stimulus with alternating polarity at 21.1 c/s and 2000 sweeps. 

SPSS version 18 was operated in 0.05 significant levels. Moreover, Colmogrov-Smirnov test was used to define normal distribution of data. To investigate the relationship between serum bilirubin levels and mean hearing thresholds, spearman correlation was used.

**Table 1:** Subjects Characteristics and Auditory Assessment Results of 18 Children

**N**	**Age (years)**	**Sex**	**TSB (mg/dL)**	**Mean behavioral thresholds (dBHL)**	**ABR**	**TEOAE**	**Prematurity**
1	5.2	Male	32	62.5	Present	Absent	No
2	10	Female	17	8.75	Present	Present	No
3	7	Female	20.8	66.7	Absent	Absent	Yes
4	3.8	Male	17	105	Absent	Absent	No
5	4.7	Female	20	83.75	Absent	Absent	No
6	5.6	Male	21	105	Absent	Absent	No
7	4.8	Male	20	98.75	Absent	Absent	No
8	11	Female	38	88.75	Absent	Absent	No
9	3	Male	17	85	Absent	Absent	No
10	3	Female	19	66.25	Present	Absent	Yes
11	4	Female	19	110	Absent	Absent	No
12	6.3	Female	20	5	Present	Present	No
13	2.4	Female	48	63.75	Present	Absent	No
14	11	Female	38	50	Present	Absent	No
15	6.2	Male	30.5	7.5	Present	Present	No
16	3.2	Male	26.8	11.25	Present	Present	No
17	5.7	Female	30.3	7.5	Present	Present	No
18	5.2	Male	32	7.5	Present	Present	No

TSB indicates total serum bilirubin level

## Results

None of the children had a history of birth hypoxia. Two cases (11.1%) had a history of premature birth (prior to 37 weeks) and both had abnormal behavioral hearing thresholds. None of the children had a history of very low birth weight (less than 1500 g). Overall, 6 subjects (33.3%) had normal behavioral hearing thresholds and 12 subjects (66.7%) had some degree of hearing loss. One of the children had moderate hearing loss, four patients with moderate to severe hearing thresholds and seven had severe to profound hearing loss.

**Fig 1:**
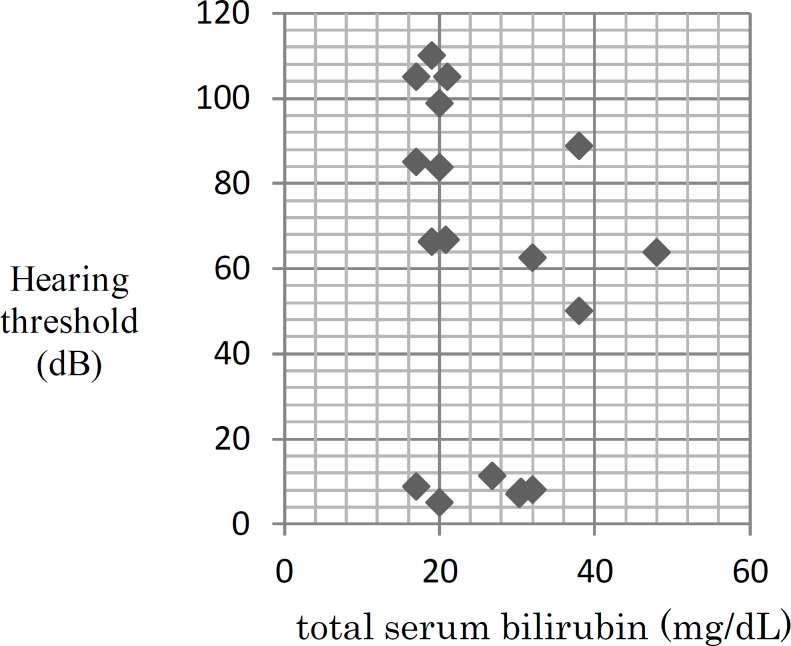
Distribution of Average Hearing Thresholds According to the Level of Bilirubin

Average behavioral hearing thresholds in four frequencies measured, standard deviations, upper bound and lower bound of thresholds, in cases with hearing impairment, had been presented in Table 2. All cases with SNHL were affected by bilateral symmetric hearing loss. Total serum bilirubin (TSB) levels of these children at neonatal period were between 17-48 mg/dL (mean 25.9±8.8 mg/dL). Phototherapy was the only treatment in the 55.6% of the children and for the remaining ones exchange transfusion was performed in addition to phototherapy. Regarding those who were transfused, one child comprised three times, another one had it done twice, and the rest had once. Figure 1 reveals distribution of serum bilirubin levels in terms of the average hearing thresholds.

**Table 2:** Mean Standard Deviation, Upper Limit and Lower Limit of Behavioral Hearing Thresholds in Hearing Impaired Children

N	Mean (dB)	Max (dB)	Min (dB)	Standard deviation	Frequency (Hz)
**23**	72.39	100	40	17.5	500
**23**	81.74	110	55	16.34	1000
**23**	88.48	120	60	17.08	2000
**22**	90.23	110	65	17.62	4000

TOAE responses were recorded bilaterally and only in children with normal hearing. Generally, in 12 ears (33.3%), there was an acceptable response. Amplitude of responses was in the range of 5.3 - 22.2 dBSPL, with a mean of 12.8 ±4.9 dB. All normal hearing children had normal ABR responses. Changes in the ABR response in hearing impaired children ranged from no detectable wave I to total absence of response. Of 12 hearing impaired subjects, ABR response was seen bilaterally in 4 patients. None of hearing impaired cases had the wave I and only waves III and V were recorded subsequently. Due to the small number of hearing impaired cases with recordable ABR response, statistical evaluation to assess the significance of absolute and interpeak latency findings was not possible. 

In hearing impaired children, the wave form was partly distorted. Wave III was seen only in one patient. History of hearing tests had been performed in these children revealed that OAE and ABR responses in most of them had been unchanged over the time. 

Only three of them had TEOAE response at birth which was removed following a few months. ABR response was not identifiable in these children at birth however, at the time of this study, ABR had been recorded.

Assessing the relationship between bilirubin levels and average hearing thresholds at four frequencies of 500, 1000, 2000 and 4000 Hz in both ears showed that there was no significant correlation between serum bilirubin levels and the mean behavioral hearing thresholds of the total sample of 18 children, 36 ears (r=-0.28, P=0.09). Furthermore, a significant correlation was not observed between behavioral hearing thresholds in cases of SNHL (12 children, 24 ears) with serum bilirubin levels (r= -0.39, P= 0.06).

## Discussion

Average hearing thresholds of children in this study had a range of normal hearing to profound hearing loss. Overall, two of the children had a history of preterm birth and in both the cases there were some degree of hearing loss. 

Bilirubin toxicity affects the performance of the auditory nerve in several ways. In general, the more central nervous system is exposed to the bilirubin, the more impact on hearing performance will occur (17). Since it is not possible to accurately determine exposure to bilirubin, its consequence on the hearing is highly variable and can range from slight hearing loss to complete deafness. Studies have shown that hearing impairment due to kernicterus is more common in premature infants who develop high bilirubin levels (17,18).

The present study showed that there is not any considerable relationship between the peak serum bilirubin levels during infancy and behavioral hearing thresholds. In other words, it is not possible to establish a direct connection between peak serum bilirubin levels and hearing thresholds. These findings are consistent with the results of previous studies on infants with a history of neonatal hyperbilirubinemia. In previous studies on neonatal jaundice, it has been reported that there is no considerable relationship between abnormalities of ABR amplitude and latency values and maximum levels of bilirubin in neonatal period (9,19). However, according to some reports, at bilirubin levels greater than 25 mg/dL there is a remarkable relationship between ABR abnormalities and maximum serum bilirubin levels (20,21). In the study of Jiang et al in 2007 on 90 infants with a history of neonatal hyperbilirubinemia, it was described that recorded thresholds and response latencies of all waves showed considerable increases and at the same time, there was no remarkable correlation between serum bilirubin concentration and increased latencies (9). Moreover, Jiang and colleagues in 2009 in a study on 83 term infants with serum bilirubin levels more than 10 mg/dL exhibited a significant decrease in the amplitude of waves III and V. However, none of the amplitude values ​​obtained had a considerable relation with total serum bilirubin levels. In explaining these findings, it should be noted that in neonates suffering from jaundice, there are many other risk factors for the occurrence of hearing loss. At the same time with increased blood bilirubin levels, damage to the hearing system might be happened. In the present study, based on the findings of the case history and in the inclusion criteria, we tried to select children who did not have any other risk factors in their registered medical history. Although, we are not confident enough that there was not presence of any other risk factors in the family history of the children and their possible role vis a vis hearing loss cannot be ruled out. Considering the fact that in the former studies which significant relationship had not been reported between serum bilirubin levels and ABR abnormalities, it is not unexpected that behavioral hearing thresholds and peak blood bilirubin levels have directly relationship together. Similarly, the results of the present study do not report a considerable relationship between the threshold values and the level of bilirubin. As reported in former studies, even moderate levels of bilirubin can also lead to the occurrence of sensorineural hearing loss (15). In general, the lack of a direct relationship between serum bilirubin levels and the average hearing thresholds suggests the importance of care for different degrees of neonatal hyperbilirubinemia, because of the risk of trauma to the auditory system.

According to Rhee and colleagues (1999), in cases of neonatal hyperbili- rubinemia, the location of hearing impairment is outside of the cochlea and cochlear function will be unaffected (13). In contrast, Sheykholeslami and Kaga (1999) and Sano et al (2005) have reported that lesions caused by increased levels of blood bilirubin may involve organ of Corti in the cochlea and especially the outer hair cells (12,15). In the present study, lack of TEOAE response in all hearing impaired children, as well as the absence of ABR response for most of them, suggests that in some cases of neonatal jaundice there is the possibility of damage to the cochlear structures besides neurological problems.

Auditory neuropathy spectrum disorder (ANSD) is defined as the presence of OAEs and/or cochlear microphonics (CMs) associated with severe ABR abnormalities or absent of ABR response (22,23). As mentioned above, three of hearing impaired children in this study had TEOAE response at birth which disappeared following a few months. There was no identifiable ABR response in these children at birth. However, at the time of this study, ABR was recorded. Considering these findings and the symptoms of ANSD, there is the possibility of this type of hearing impairment in the history of the children. Recovery of ANSD symptoms and improved ABR response is a finding that has been reported in former studies (24,25). However, none of the hearing impaired cases and those with normal hearing thresholds, had symptoms of the ANSD. These findings indicate that in abnormalities similar to auditory neuropathy spectrum disorder, which perceived following neonatal hyperbilirubinemia, there is a possibility of physiological changes in the auditory system. Therefore, monitoring of hearing status over time in these children would be essential.

The findings of this study increase knowledge of the effects of neonatal hyperbilirubinemia on hearing. Inability to provide appropriate services to reduce dangerous levels of bilirubin can lead to chronic and permanent hearing impairment which has been reported in almost 10 percent of children with a history of neonatal hyperbilirubinemia (7). Using the results of our study and similar studies would provide practical messages for physicians, parents and laymen regarding effects of neonatal hyperbilirubinemia on the auditory system and the prompt remedial action may condense possible consequences of neonatal jaundice.

## Conclusions

The present study showed that increased blood bilirubin levels in the neonatal period can be considered as one of the causes of hearing loss. Based on our findings, neonatal hyperbilirubinemia can cause abnormalities in auditory evaluations, including behavioral audiometry, without developing kernicterus. However, it seems that there is not any clear and direct relationship between serum bilirubin levels and hearing thresholds. Therefore, behavioral hearing thresholds and hearing system impairment cannot be estimated solely based on maximum bilirubin levels at birth. 
